# Supplementary material for: Anti-Inflammatory Salidroside Delivery from Chitin Hydrogels for NIR-II Image-Guided Therapy of Atopic Dermatitis
Source: J Funct Biomater. 2023 Mar 8;14(3):150. doi: 10.3390/jfb14030150 (PMC10058600; doi:10.3390/jfb14030150)
Supplement: Supplementary file 1 [file jfb-14-00150-s001.zip › jfb-2137895-supplementary.pdf]

Supplementary Materials

Anti-Inflammatory Salidroside Delivery from Chitin Hydrogels for NIR-II Image-Guided Therapy of Atopic Dermatitis

Shengnan He [1,†], Fang Xie [2,†], Wuyue Su [1], Haibin Luo [3], Deliang Chen [4], Jie Cai [2,5,*] and Xuechuan Hong [1,6,*]

1 State Key Laboratory of Virology, College of Science, Research Center for Ecology, Laboratory of Extreme Environmental Biological Resources and Adaptive Evolution, Medical College, Tibet University, Lhasa 850000, China
2 Hubei Engineering Centre of Natural Polymers-Based Medical Materials, College of Chemistry & Molecular Sciences, Wuhan University, Wuhan 430072, China
3 Key Laboratory of Tropical Biological Resources of Ministry of Education, School of Pharmaceutical Sciences, Hainan University, Haikou 570228, China
4 Jiangxi Key Laboratory of Organo-Pharmaceutical Chemistry, Chemistry and Chemical Engineering College, Gannan Normal University, Ganzhou 341000, China
5 Institute of Hepatobiliary Diseases, Zhongnan Hospital of Wuhan University, Wuhan 430071, China
6 Wuhan University Shenzhen Research Institute, Shenzhen 518057, China
* Correspondence: caijie@whu.edu.cn (J.C.); xhy78@whu.edu.cn (X.H.)
† These authors contributed equally to this work.

1. DPPH

The experimental group（A0）consisted of 0.04 mg/mL DPPH solution and 100 μL of QCOD@Sal at 10, 5, 2.5 and 1.25 mg/mL concentrations. The control group (A_j) was 100 μL of absolute ethanol solution and 100 μL of QCOD@Sal at 10, 5, 2.5 and 1.25 mg/mL concentrations. The standard group (A_i) was 100 μL of absolute ethanol solution and 100 μL of 0.04 mg/mL DPPH solution. Each group was incubated for 30 min at 517 nm, and the absorbance was measured and the clearance was calculated.

$$\text{DPPH scavenging (\%)} = [1-(A_0- A_j)/ A_i] \times 100$$

2. Preparation of NIR-II probes

To a solution of compound **HLA4-TMS** (10 mg, 0.0157 mmol) in DCM (2 mL) was added TFA (1 mL) at 0 ^{0}C. The reaction mixture was slowly warmed to room temperature. TLC analysis indicated that the reaction was completed within 8 h. The reaction mixture was concentrated *in vacuo* and the crude product **HLA4** was used for the next step without

further purification. To the solution of compound **HLA4** (1.3 mg, 0.0025 mmol) in anhydrous DMF (900 μL) was added 10 μL DIPEA under N_2 atmosphere. Then *N*-(2-Aminoethyl) maleimide trifluoroacetate salt (0.7611 mg, 0.0030 mmol) and HATU (1.1410 mg, 0.0030 mmol) were added into the solution. The reaction was kept at 25 °C overnight. The reaction mixture was concentrated *in vacuo*. The crude product was purified by silica gel chromatography (dichloromethane: methanol = 10:1 v/v) to afford a green soild **HLA4P** (20 mg, 54% yield).

3. Controlled release of salidroside in vitro

In detail, firstly, different concentrations of salidroside aqueous solutions (9.375, 12.5, 15.625, 25, 31.25, 50, 62.5, 83.33, 125, 250 μg/mL) were prepared, and then the absorbance at 275 nm was measured by UV spectrophotometer, and finally the absorbance values and solution concentrations were used as the horizontal and vertical coordinates to make the curves and perform The standard curve.

4. Hemolysis experiment

First, the whole blood of mice was taken and centrifuged for 10 min at 1000 rpm. After centrifugation, the supernatant was washed with PBS (15 mL × 3) until the supernatant was no longer red and cloudy after centrifugation. The concentration of 5 % (v/v) erythrocyte suspension was obtained by diluting the deposited erythrocytes with PBS. The hydrogel was mixed with the red blood cells (1:1, v/v). The experiment was divided into four groups: the negative control group, the positive control group, the QCOD group, and the QCOD@Sal group. The negative control group consisted of 200 microliter PBS buffer and 200 microliter erythrocyte suspension. The positive control group consisted of 200 microliters of primary water and 200 microliters of erythrocyte suspension. The QCOD group consisted of 200 microliter QCOD hydrogel and 200 microliter erythrocyte suspension. The QCOD@Sal group consisted of 200 microlitre QCOD@Sal and 200 microlitre erythrocytes. After mixing evenly, the supernatant was centrifuged and placed in a 96-well plate with 100 microliters per well and 4 multiple Wells for each sample. Measure the absorbance wavelength at 540 nm.

5. Blood routine examination

On the 15th day of the experiment, the blood of mice (orbital blood) was taken by 0.35 mm capillary glass tube infiltrated with 100 IU/mL sodium heparin solution for 1 h and dried). 5 drops of blood were taken from the orbit of mice into 1 ml of anticoagulant tube containing EDTA, shaken well and then put into the automatic blood biochemical analysis instrument (Tercom Veterinary Automatic Blood Cell Analyzer, TEK-VET3) to determine the content and percentage of whole blood leukocytes and their classification. GraphPad Prism 8 software was used for analysis, and differences between groups were analyzed by t-test, with $p^* < 0.05$ as a significant difference.

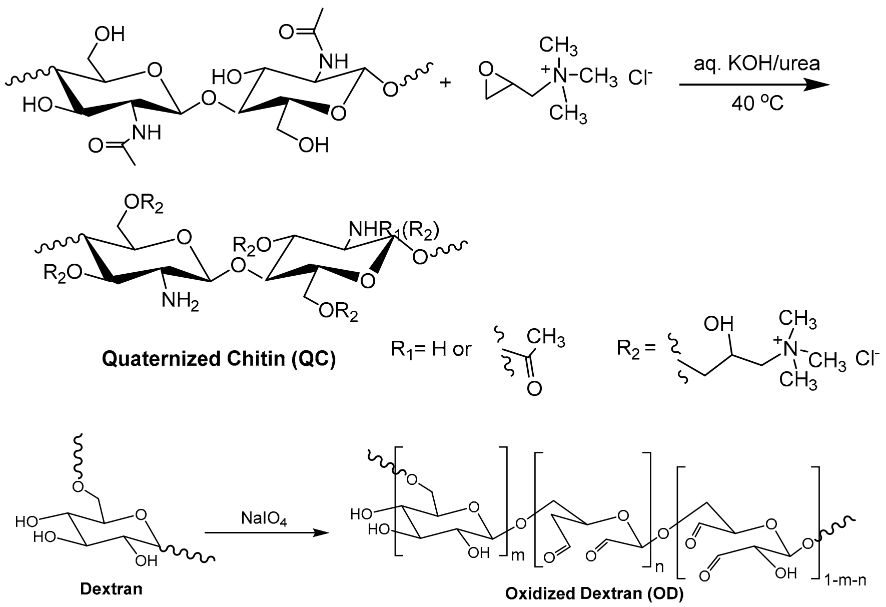

Figure S1. QC homogeneously synthesized in KOH/urea aqueous solution. The synthetic route of dextran CHO.

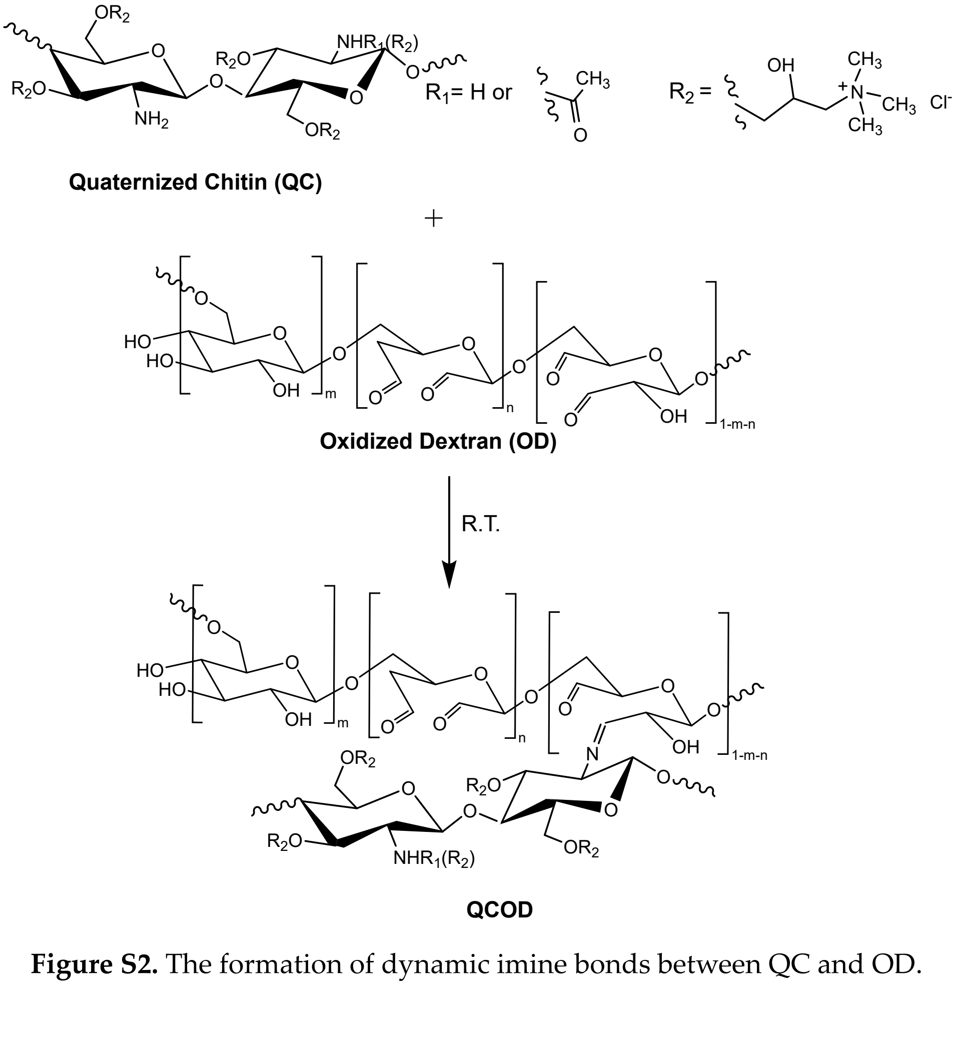

Figure S2. The formation of dynamic imine bonds between QC and OD.

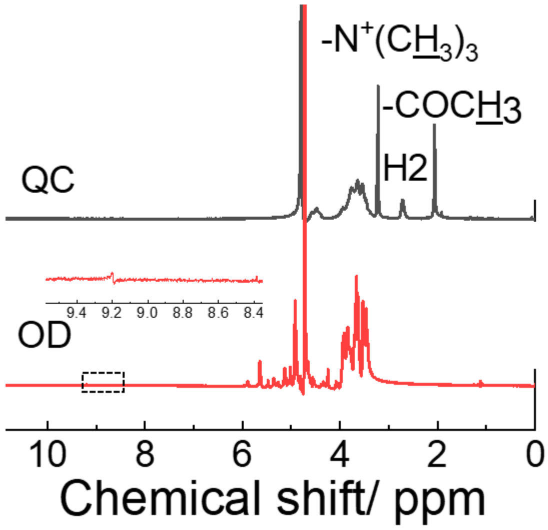

Figure S3. ^{1}H NMR spectra of QC and dextran-CHO (OD).

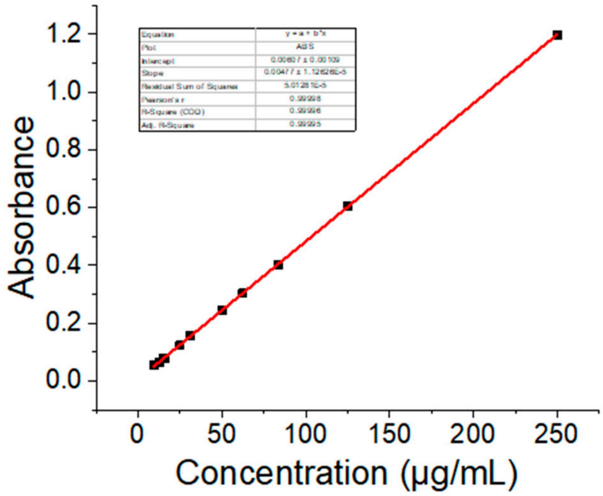

Figure S4. The controlled release of salidroside *in vitro.*

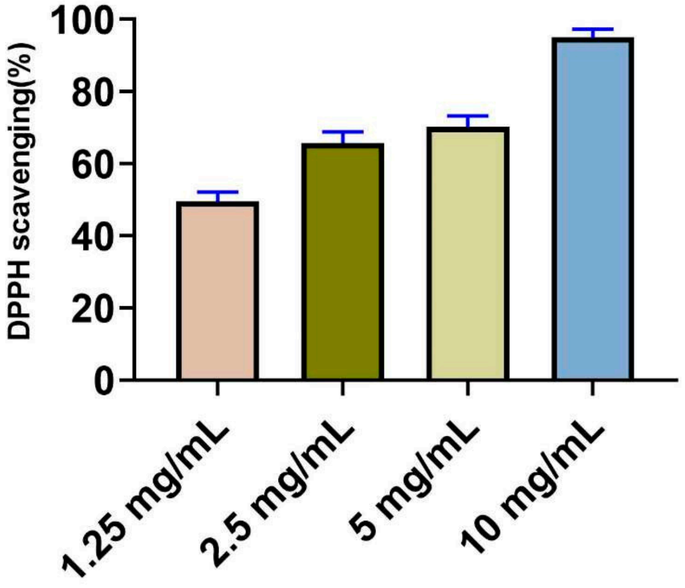

Figure S5. Antioxidant efficiency of salidroside hydrogels.

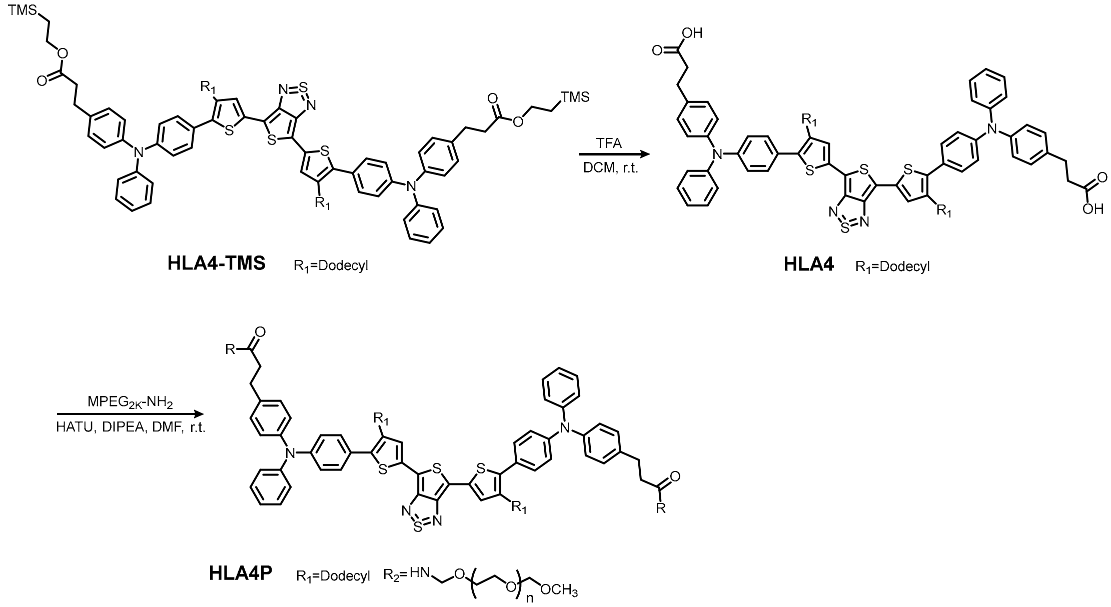

Figure S6. The preparation of HLA4P.

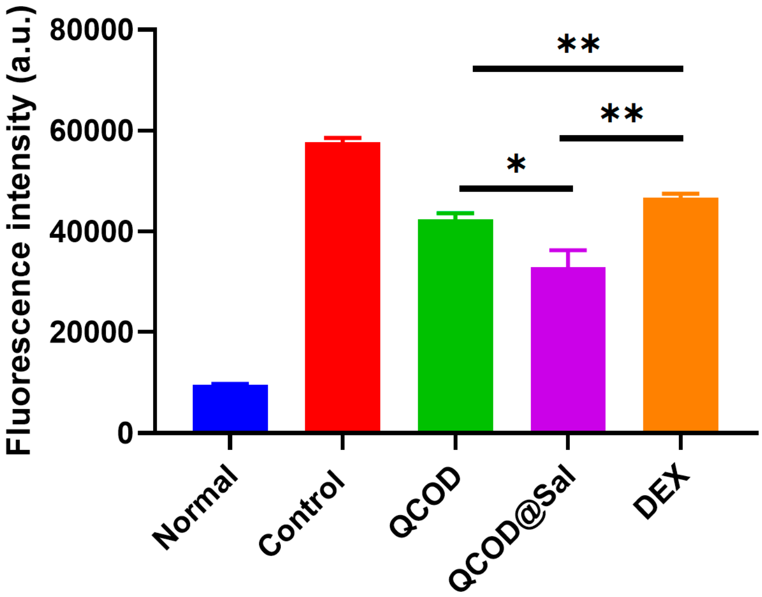

Figure S7. The fluorescence intensity (FL. intensity).

As shown in Fig S7, the fluorescence signal intensity of the QCOD@Sal treatment group was significantly lower than that of the other two treatment groups, and the therapeutic effect of QCOD@Sal was significantly stronger than that of the DEX treatment group compared with the positive drug DEX.
